# Spectrum of Papillary Breast Lesions According to World Health Organization Classification of Papillary Neoplasms of Breast

**DOI:** 10.7759/cureus.11026

**Published:** 2020-10-18

**Authors:** Atif A Hashmi, Mahrukh Faraz, Sana Rafique, Hiba Adil, Abira Imran

**Affiliations:** 1 Pathology, Liaquat National Hospital and Medical College, Karachi, PAK; 2 Internal Medicine, Liaquat National Hospital and Medical College, Karachi, PAK; 3 Internal Medicine, Memon Medical Institute Hospital, Karachi, PAK; 4 Statistics, Liaquat National Hospital and Medical College, Karachi, PAK

**Keywords:** papillary breast lesions, intraductal papilloma, papilloma with atypical ductal hyperplasia (adh), papilloma with ductal carcinoma in situ (dcis), papillary dcis, solid papillary carcinoma in situ, solid papillary carcinoma with invasion, invasive solid papillary carcinoma, encapsulated papillary carcinoma, encapsulated papillary carcinoma with invasion

## Abstract

Introduction

Papillary breast lesions are segregated into benign and malignant based on the presence or absence of myoepithelial cells in the papillary cores. Papillary breast lesions are further classified into: intraductal papilloma, papilloma with atypical ductal hyperplasia (ADH)/ductal carcinoma in situ (DCIS), papillary DCIS, solid papillary carcinoma in situ, solid papillary carcinoma with invasion, invasive solid papillary carcinoma, encapsulated papillary carcinoma and encapsulated papillary carcinoma with invasion. In this study, we evaluated the spectrum of papillary breast lesions in resection specimens of the breast according to the latest World Health Organization (WHO) classification of breast tumors.

Methods

This was a retrospective cross-sectional study, and was conducted at Liaquat National Hospital for a period of six years, from January 2012 till December 2017. Data of patients that underwent surgeries for breast tumors were included in the study. All specimens were grossed, according to defined protocols, and representative sections were taken after inking resection margins. Hematoxylin and eosin-stained sections were examined by experienced histopathologists, and myoepithelial stains (p63 and myosin) were done in selected sections of all tumors. Histopathological classification of papillary tumors was performed according to WHO classification of breast tumors.

Results

The study involved 190 excision specimens of papillary breast lesions. Mean age of the patients was 45.6±17.1 years. Most of the lesions were between two and five centimetres (69.1%). For invasive carcinomas (n = 76), the most frequent grade was II (52.6%). For in situ and invasive carcinomas (n = 129), lymphovascular invasion and axillary metastasis were noted in 5.4% and 9.3% cases, respectively. Among papillary breast lesions, 36.8% were benign (intraductal papilloma, solitary or multiple) while 63.2% harbored ADH, DCIS, or invasive carcinoma. Invasive papillary carcinoma was the most frequent malignant papillary lesion (20%), followed by solid papillary carcinoma with invasion (12.6%). We found significant associations between patient’s age and tumor size with histological type of papillary lesion as benign papillary lesions had smaller size and younger age compared to malignant papillary lesions.

Conclusion

We noted a high frequency of malignancy in papillary breast lesions. Moreover, malignant papillary lesions were significantly associated with higher age and larger tumor size.

## Introduction

Breast cancer is the most preponderant malignancy in women, and is especially frequent in Southeast Asia [1,2]. The high frequency of breast cancer in young women, and its poor prognosis is principally concerning [3-5]. An entrancing aspect of breast pathology involves papillary breast lesions. They are defined by the presence of fibro-vascular cores surrounded by cells [6]. Besides invasive papillary carcinoma, which is rare, most of the papillary breast lesions are intraductal. Papillary breast lesions are segregated into benign and malignant based on the presence or absence of myoepithelial cells in the papillary cores [7]. Papillary breast lesions are further classified into intraductal papilloma, papilloma with atypical ductal hyperplasia (ADH), papilloma with ductal carcinoma in situ (DCIS), papillary DCIS, solid papillary carcinoma in situ, solid papillary carcinoma with invasion, invasive solid papillary carcinoma, encapsulated papillary carcinoma and encapsulated papillary carcinoma with invasion. Pathological diagnosis of papillary breast lesions on core needle biopsies is challenging, and definitive diagnosis is only possible on resection specimens. Immunohistochemical (IHC) stains for myoepithelial markers are especially useful in these circumstances. Classification of papillary breast lesions is evolving and therefore exact frequency of different types of papillary lesions in our population is unknown. Therefore, in this study, we evaluated the spectrum of papillary breast lesions in resection specimens of the breast according to the latest world health organization (WHO) classification of breast tumors.

## Materials and methods

This was a retrospective cross-sectional study, and was conducted at Liaquat National Hospital for a period of six years, from January 2012 until December 2017. Data of patients that underwent surgeries for breast tumors were included in the study. All cases that were diagnosed as primary papillary breast lesions on resection were included in the study. All these cases had a preoperative diagnosis of papillary breast lesion (with or without atypia) on trucut biopsy. Cases with a diagnosis of invasive ductal carcinoma or any other breast tumor were excluded from the analysis. In addition, cases with a history of pre-operative chemo-radiation were also excluded. Specimens included lumpectomies/breast conservation surgeries, microdochetomy specimens, simple mastectomy, and modified radical mastectomies. All specimens were grossed, according to defined protocols, and representative sections were taken after inking resection margins. Hematoxylin and eosin-stained sections were examined by experienced histopathologists, and myoepithelial stains (p63 and myosin) were done in selected sections of all tumors. Histopathological classification of papillary tumors was performed according to WHO classification of breast tumors.

Data analysis was performed using Statistical Package for Social Sciences (SPSS, Version 26.0; IBM Inc., Armonk, NY, USA). Chi square test was used to check the association. P-values ≤ 0.05 were considered as significant.

## Results

The study involved 190 excision specimens of papillary breast lesions. Mean age of the patients was 45.6±17.1 years. Majority of the patients were over 50 years of age (39.5%). Most of the lesions were between two and five centimetres (69.1%). For invasive carcinomas (n = 76), the most frequent grade was II (52.6%). For in situ and invasive carcinomas (n = 129), lymphovascular invasion and axillary metastasis were noted in 5.4% and 9.3% cases, respectively, as shown in Table 1.

**Table 1:** Descriptive statistics of the population under study MRM, modified radical mastectomy

Characteristic	Frequency (%)
Age	
<30 years	42 (22.1)
30–50 years	73 (38.4)
>50 years	75 (39.5)
Specimen type	
Lumpectomy	152 (80)
Simple mastectomy	13 (6.8)
MRM	23 (12.1)
Tumor Size	
<2	50 (26.3)
2–5	118 (62.1)
>5	22 (11.6)
Grade (n = 76)	
I	13 (17.1)
II	40 (52.6)
III	23 (30.3)
Lymphovascular invasion (n = 129)	
Present	7 (5.4%)
Absent	122 (94.6%)
Axillary metastasis (n = 129)	
Present	12 (9.3%)
Absent	117 (90.7%)

**Figure 1:**
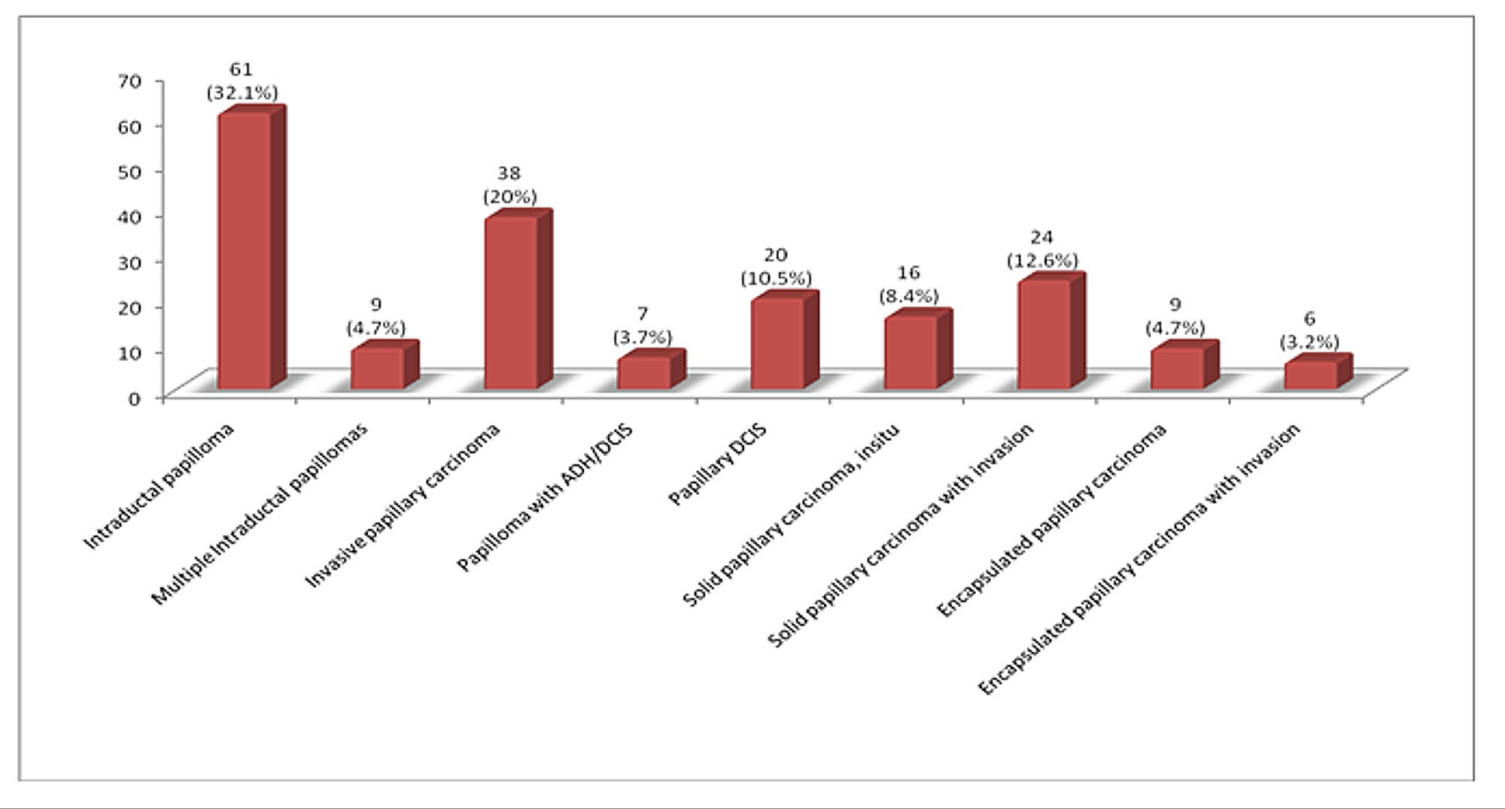
Frequency distribution of papillary breast lesions DCIS, Ductal carcinoma in situ

Among papillary breast lesions, 36.8% were benign (intraductal papilloma, solitary or multiple) while 63.2% harbored ADH, DCIS or invasive carcinoma. Invasive papillary carcinoma was the most frequent malignant papillary lesion (20%), followed by solid papillary carcinoma with invasion (12.6%) as shown in Figure 1.

We found significant associations between patient’s age and tumor size with histological type of papillary lesion as benign papillary lesions had smaller size and younger age compared to malignant papillary lesions (Table 2).

**Table 2:** Association of patents’ age and tumor size with histologic type of papillary lesion Chi square test was applied * p-value significant as  <0.05 ADH, atypical ductal hyperplasia; DCIS, ductal carcinoma in situ

Histologic type of papillary lesion		Intraductal papilloma	Multiple intraductal papillomas	Invasive papillary carcinoma	Papilloma with ADH/DCIS	Papillary DCIS	Solid papillary carcinoma, in situ	Solid papillary carcinoma with invasion	Encapsulated papillary carcinoma	Encapsulated papillary carcinoma with invasion	P-value
Age	<30 years	37 (60.7)	5 (55.6)	0 (0)	0 (0)	0 (0)	0 (0)	0 (0)	0 (0)	0 (0)	<0.001*
	30–50 years	21 (34.4)	4 (44.4)	13 (34.2)	4 (57.1)	7 (35)	6 (37.5)	9 (37.5)	6 (66.7)	3 (50)	
	>50 years	3 (4.9)	0 (0)	25 (65.8)	3 (42.9)	13 (65)	10 (62.5)	15 (62.5)	3 (33.3)	3 (50)	
Size	<2 cm	32 (52.5)	1 (11.1)	3 (7.9)	3 (42.9)	4 (20)	1 (6.3)	4 (16.7)	2 (22)	0 (0)	<0.001*
	2–5 cm	28 (45.9)	7 (77.8)	27 (71.1)	4 (57.1)	15 (75)	11 (68.8)	16 (66.7)	5 (55.6)	5 (83.3)	
	>5 cm	1 (1.6)	1 (11.1)	8 (21.1)	0 (0)	1 (5)	4 (25)	4 (16.7)	2 (22.2)	1 (16.7)	

For in situ and invasive carcinomas, no significant association of tumor grade, lymphovascular invasion and axillary metastasis was noted with histologic type of papillary carcinoma (Table 3).

**Table 3:** Association of grade, lymphovascular and axillary metastasis with histologic type of papillary carcinoma Chi square test is applied. **p-value insignificant as >0.05 DCIS, ductal carcinoma in situ; N/A, Not applicable

Histologic type of papillary carcinoma		Invasive papillary carcinoma	Papillary DCIS	Solid papillary carcinoma, in situ	Solid papillary carcinoma with invasion	Encapsulated papillary carcinoma	Encapsulated papillary carcinoma with invasion	P-value
Grade	I	2 (5.4)	N/A	N/A	7 (29.2)	N/A	0 (0)	<0.236**
	II	14 (37.8)	N/A	N/A	16 (66.7)	N/A	6 (100)	
	III	21 (56.8)	N/A	N/A	1 (4.2)	N/A	0 (0)	
Lymphovascular invasion	Present	3 (7.9)	0 (0)	0 (0)	2 (8.3)	1 (11.1)	1 (16.7)	0.359**
	Absent	35 (92.1)	20 (100)	16 (100)	22 (91.7)	8 (88.9)	5 (83.3)	
Axillary metastasis	Present	7 (18.4)	0 (0)	1 (6.3)	2 (8.3)	1 (11.1)	1 (16.7)	0.282**
	Absent	31 (81.6)	20 (100)	15 (93.8)	22 (91.7)	8 (88.9)	5 (83.3)	

## Discussion

**Figure 2:**
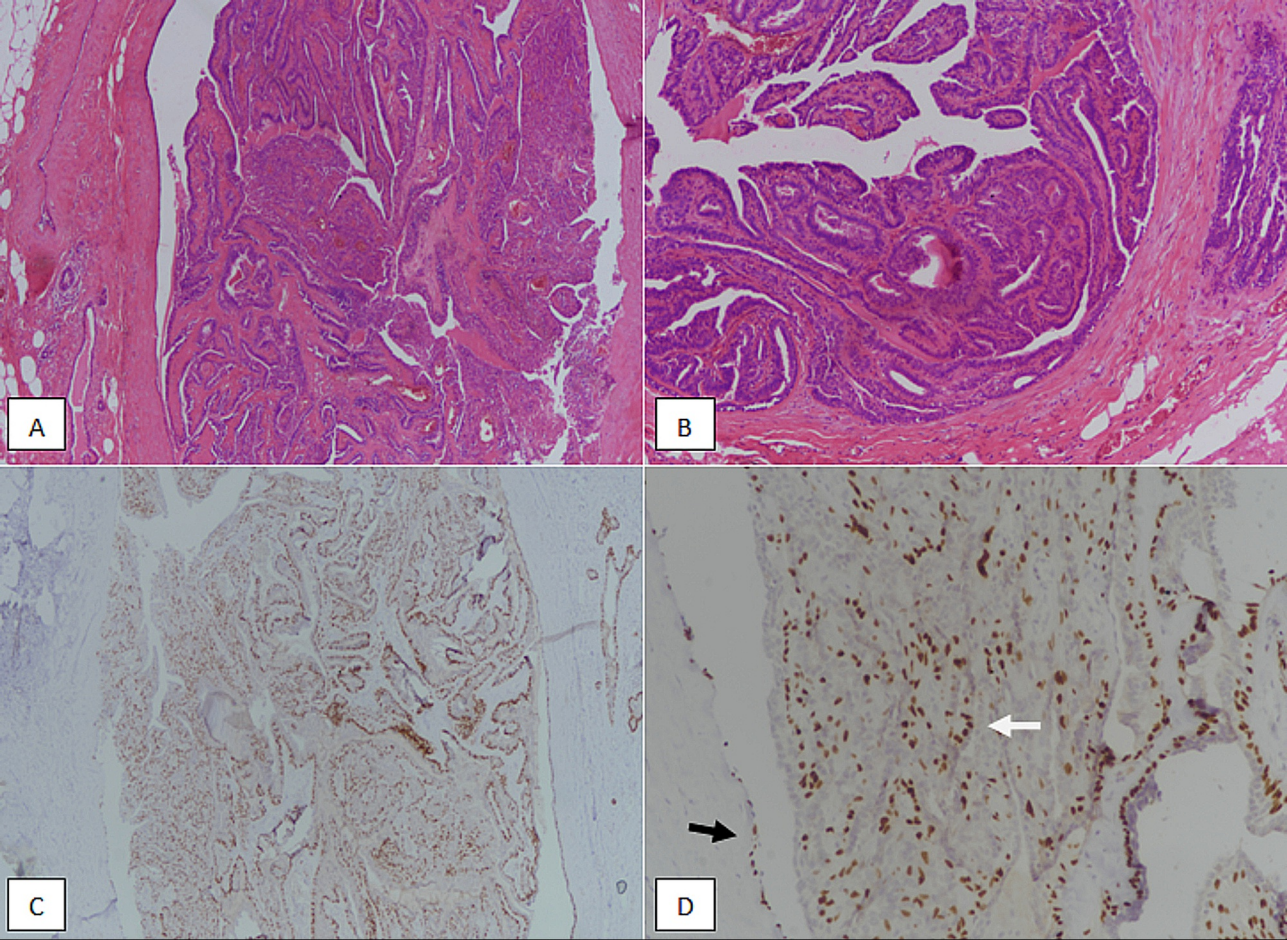
Intraductal papilloma. (A): H & E section at 100X magnification showing intraductal papillary proliferation with bland features. (B): H & E section at 200X magnification showing lack of solid intraductal proliferation. (C): p63 immunostain at 100X magnification & (D): p63 immunostain at 200X magnification showing intact myoepithelial staining both at the periphery (black arrow) and centre of papillary cores (white arrow). H & E, Hematoxylin and eosin

Intraductal papilloma is the most common papillary breast lesion. Intraductal papilloma has two sub-categories: central and peripheral intraductal papilloma, depending upon the location and clinical presentation [8]. Central intraductal papilloma presents as nipple discharge. The presence of papillary clusters of epithelial cells on cytological examination of nipple discharge specimens help in differentiation from duct ectasia. Histologically, intraductal papillomas are characterized by fibrovascular cores surrounded by epithelial cells. The presence of myoepithelial cells in cores is the distinguishing feature of intraductal papilloma (Figure 2).

Intraductal papilloma can undergo a variety of pathological alterations like usual ductal hyperplasia (UDH), sclerosing adenosis, and papillary apocrine metaplasia that make diagnosis on core needle biopsy challenging. Surgical management of intraductal papilloma diagnosed on core biopsy is controversial. Some authors have suggested that intraductal papilloma greater than five millimetres should be excised [9]. In our study, the majority of intraductal papillomas were less than five centimetres (52.5%), and more than 90% were solitary.

**Figure 3:**
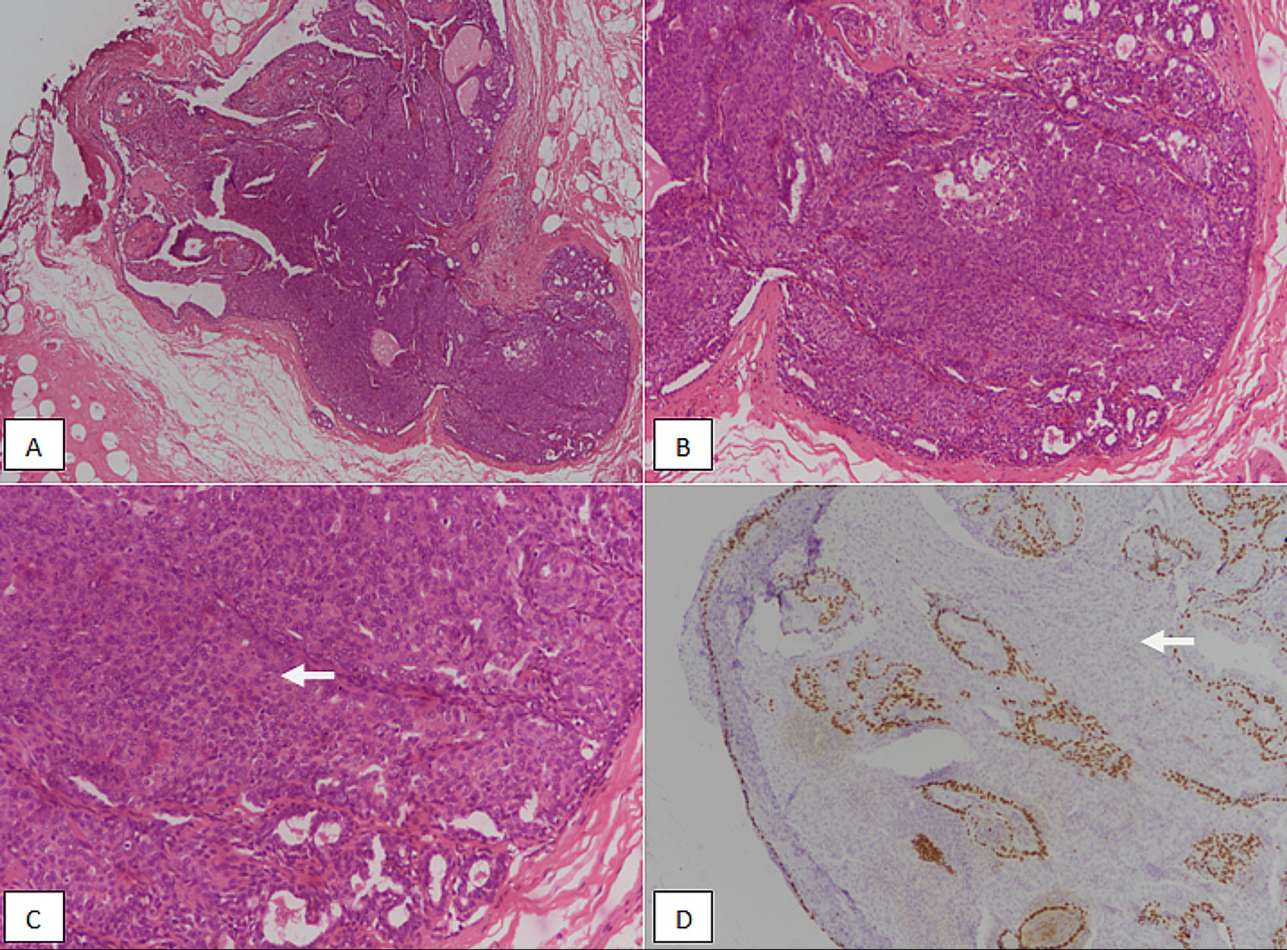
Intraductal papilloma with DCIS. (A): H & E stained section at 100X magnification showing intraductal proliferation with solid areas. (B): H & E staining at 200X magnification. (C): 400X magnification revealing foci of solid proliferation with features of low grade DCIS (arrow). (D): p63 immunostain with solid areas showing loss of p63 staining (arrow). H & E, Hematoxylin and eosin; DCIS, Ductal carcinoma in situ

Intraductal papilloma sometimes harbors atypical epithelial proliferation, referred to as intraductal papilloma with ADH, or intraductal papilloma with DCIS. This diagnosis should only be rendered when atypical proliferation meets the criteria of low grade DCIS outside the context of papilloma as shown in Figure 3.

Differentiation of papilloma with ADH and DCIS is based on size cutoff of three millimetres [10]. Richter-Ehrenstein et al. investigated 61 excision specimens of intraductal papilloma and found six of them atypical, five with DCIS, and two with invasive carcinoma [11]. We had 77 cases of intraductal papilloma in total, out of which seven cases (0.9%) had foci of ADH/DCIS.

**Figure 4:**
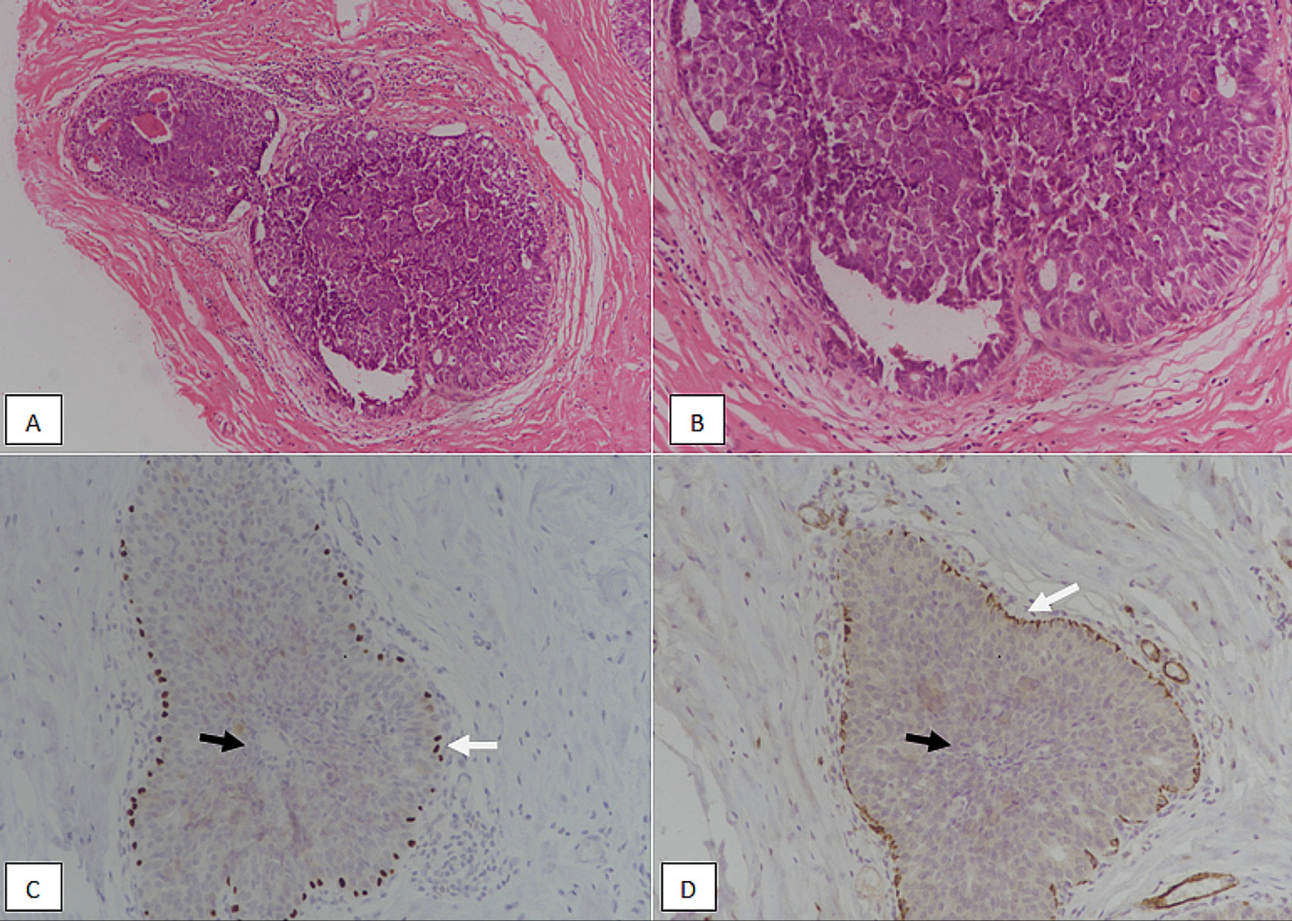
Papillary DCIS. (A): H & E stained section at 100X magnification showing atypical intraductal proliferation with papillary cores and features of low grade DCIS. (B): H & E staining at 200X magnification. (C): p63 immunostain showing loss of nuclear myoepithelial staining in the centre of papillary cores (black arrow) and intact nuclear myoepithelial staining at the periphery (white arrow). (D): Myosin immunostain showing loss of cytoplasmic myoepithelial staining in the centre of papillary cores (black arrow) and intact cytoplasmic staining at the periphery (white arrow). H & E, Hematoxylin and eosin; DCIS, Ductal carcinoma in situ

Papillary DCIS is an atypical intraductal proliferation with fibrovascular cores devoid of myoepithelial layer in the central cores, while the myoepithelial layer is maintained at the periphery (Figure 4).

Papillary DCIS can be exclusive; however, it is commonly associated with other patterns of DCIS or invasive ductal carcinoma. In our study, 10% of papillary lesions were papillary DCIS in the absence of invasive carcinoma.

**Figure 5:**
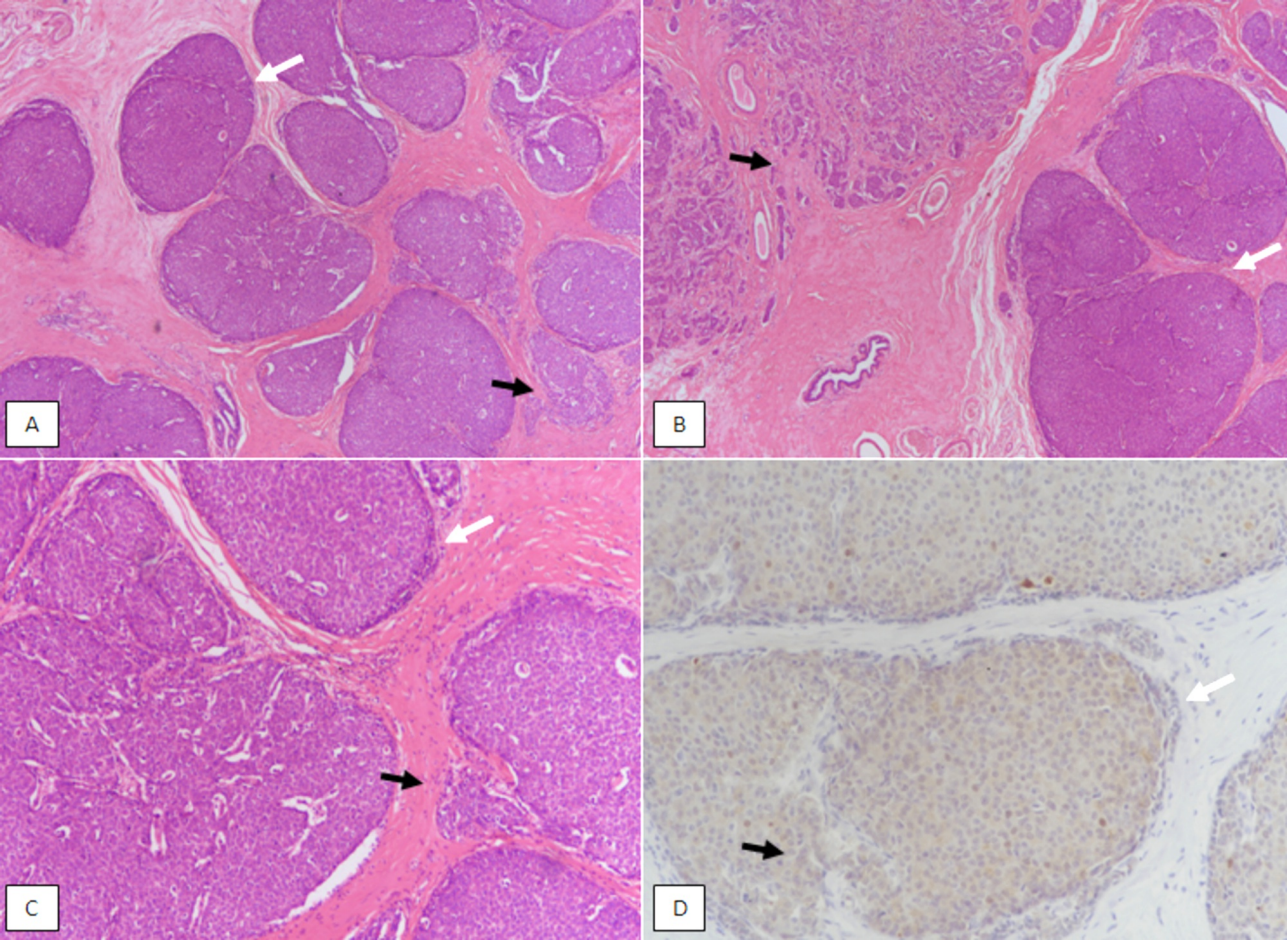
Solid papillary carcinoma. (A) & (B): H & E stained sections at 100X magnification showing discrete solid nodules of tumor with indistinct papillary cores. Foci of solid papillary carcinoma in situ show sharp circumscribed borders (white arrow). Foci of invasion are also noted with irregular borders (black arrow). (C): H & E staining at 200X magnification revealing in situ solid papillary carcinoma with obliterated fibrovascular cores and circumscribed borders (white arrow) and invasive foci with irregular edges (black arrow). (D): p63 immunostain showing loss of nuclear myoepithelial staining both in the centre of papillary cores (black arrow) and at the periphery (white arrow). H & E, Hematoxylin and eosin

Solid papillary carcinoma is characterized by multiple solid nodules of low-grade epithelial proliferation, with delicate fibrovascular network. It can sometimes be confused with cribriform carcinoma or cribriform pattern DCIS. Solid papillary carcinomas mostly lack myoepithelial layer, both in the cores and at the periphery (Figure 5).

Solid papillary carcinoma can be in situ or invasive. The invasive component derives the pathological TMN stage. A morphological review of 108 cases of solid papillary carcinoma showed that solid papillary carcinomas were associated with better prognostic features compared to invasive ductal carcinoma [12]. In our study, there were 40 cases of solid papillary carcinoma, out of which, 24 (60%) had invasive component. The majority of patients with solid papillary carcinoma were more than 50 years of age and only three cases (7.5%) revealed axillary metastasis signifying prognostically good pathological features.

**Figure 6:**
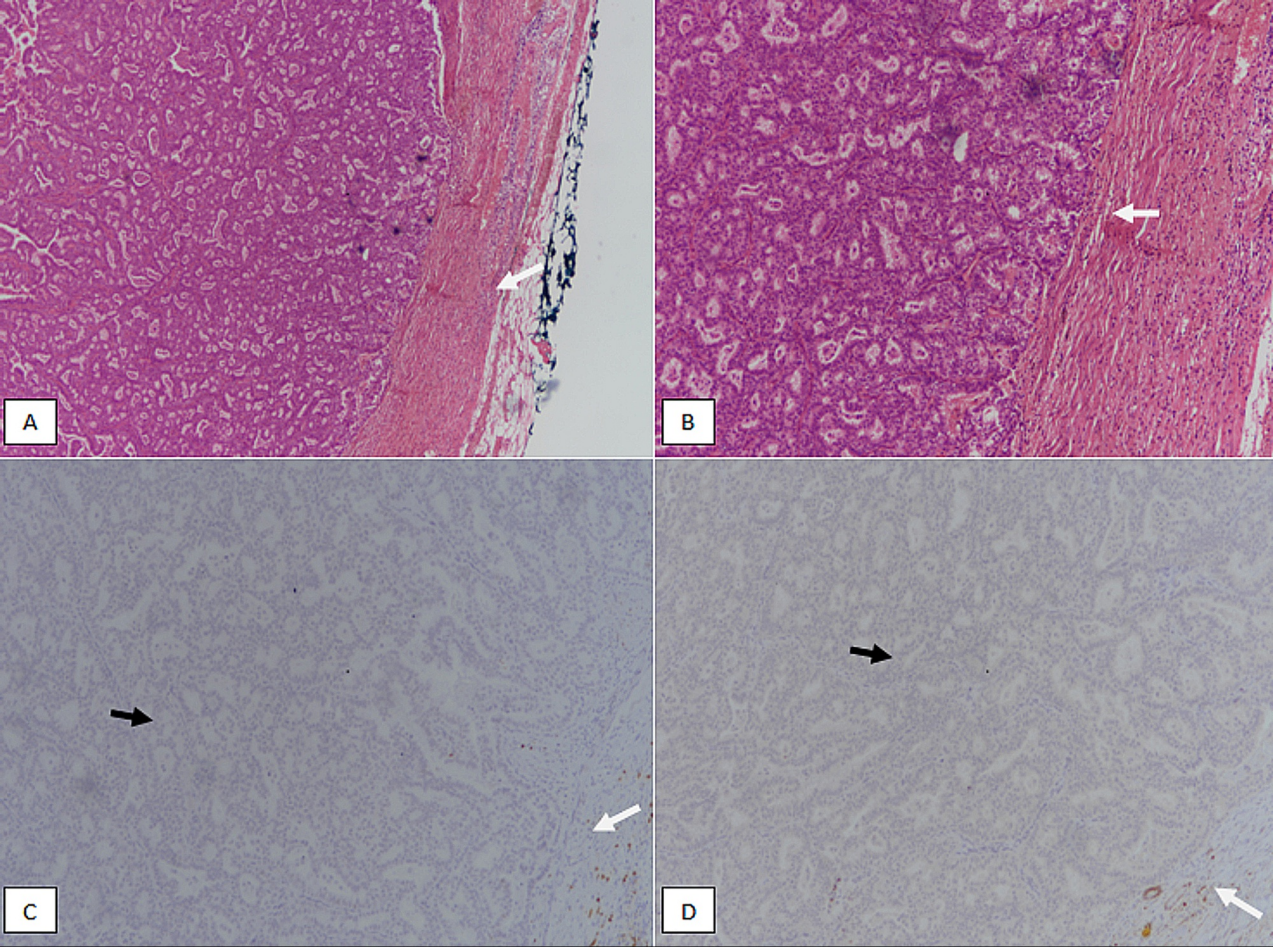
Encapsulated papillary carcinoma. (A): H & E stained section at 100X magnification showing circumscribed atypical proliferation of epithelial cells with indistinct papillary cores and surrounded by a fibrous capsule (white arrow). (B): H & E staining at 200X magnification revealing circumscribed borders of the tumor (white arrow). (C): p63 immunostain showing loss of nuclear myoepithelial staining both in the centre of papillary cores (black arrow) and at the periphery (white arrow). (D): Myosin immunostain showing loss of cytoplasmic myoepithelial staining both in the centre of papillary cores (black arrow) and at the periphery (white arrow). H & E, Hematoxylin and eosin

Encapsulated papillary carcinoma has a thick fibrous core at the periphery. Fibrovascular cores are thin and delicate, lined by atypical epithelial cells. Similar to solid papillary carcinoma, encapsulated papillary carcinoma mostly lacks myoepithelial layer in both the center of fibrovascular cores and at the periphery (Figure 6).

Encapsulated papillary carcinoma with invasion should only be diagnosed if the invasive component is outside the fibrous capsule, and is mostly invasive ductal carcinoma. We had 15 cases of encapsulated papillary carcinoma, out of which six (40%) cases had invasive component. In a study involving 19 cases of encapsulated papillary carcinoma found that nine cases were associated with invasive ductal carcinoma [13].

Invasive papillary carcinoma is a rare infiltrating malignant tumor that is characterized by papillary clusters of atypical cells with background desmoplastic reaction. We found a high frequency of invasive papillary carcinoma (20%), which is high compared to existing literature.

We acknowledge a few limitations to our study. First, the long term follow-ups of malignant papillary lesions were not available. Second, a comparison of malignant papillary lesions with invasive ductal carcinoma was not performed to determine the prognostic difference between the two. Moreover, biomarker studies including estrogen receptor (ER), progesterone receptor (PR) and human epidermal growth factor receptor-2 (HER2/neu) were not evaluated in our study. Therefore, we recommend that future studies on papillary breast lesions in our population should include these evaluations.

## Conclusions

WHO classification of papillary breast lesions is an outcome-based classification of breast papillary tumors; therefore, it is important to categorize all papillary breast lesions according to this classification. Although intraductal papilloma constitutes a vast majority of papillary breast lesions, we found a high frequency of atypical/malignant papillary breast lesions, invasive papillary carcinoma and solid papillary carcinoma with invasion were the most frequent. Hence, identifying histologic features of these tumors on core needle biopsy is tremendously important. Moreover, we also found that malignant papillary lesions were significantly associated with higher age and larger tumor size.
